# 2D Atomic Templating for the Large-Scale Synthesis of Metastable CuInS_2_ and Its Heterojunctions

**DOI:** 10.1021/acsami.5c07759

**Published:** 2025-07-24

**Authors:** Jui-Teng Chang, Yu-Xiang Chen, Hao-Ting Chin, Ding-Rui Chen, Jian-Jhang Lee, Chia-Yi Wu, Yi-Chia Chou, Mario Hofmann, Ya-Ping Hsieh

**Affiliations:** † Department of Physics, 33561 National Taiwan University, Taipei 10617, Taiwan; ‡ 71556 Institute of Atomic and Molecular Sciences, Academia Sinica, Taipei 10617, Taiwan; § International Graduate Program of Molecular Science and Technology, National Taiwan University, Taipei 10617, Taiwan; ∥ Molecular Science and Technology Program, Taiwan International Graduate Program, Academia Sinica, Taipei 10617, Taiwan; ⊥ Department of Electronic Engineering, 34900 Chung Yuan Christian University, Taoyuan 32023, Taiwan; # Department of Materials Science and Engineering, National Taiwan University, Taipei 10617, Taiwan

**Keywords:** 2D materials, CuInS_2_, atomic templating, CuAu-type wurtzite, photodetector, thermoelectric

## Abstract

CuInS_2_ has emerged as a promising material for sustainable energy technologies due to its combination of attractive electronic, economic, and ecological properties. However, current synthesis routes exhibit limited phase uniformity and degraded optoelectronic performance due to the complex Cu–In–S phase diagram. In this work, we report a synthesis strategy that overcomes these limitations and can produce CuInS_2_ with a singular crystalline phase. An atomic templating approach is described that retains the structural order of a two-dimensional (2D) transition-metal monochalcogenide host to realize phase-pure wurtzite CuAu-type CuInS_2_. Structural and spectroscopic characterization confirms the exclusive formation of this metastable phase and provides insights into its electronic properties. The transformation proceeds via a strain-mediated layer-by-layer cation-exchange mechanism, enabling precise control over its extent and interfaces. This approach establishes a generalizable route for synthesizing complex 2D heterostructures, and we demonstrate the direct growth of ternary 2D heterojunctions at large scale. The potential of this capability was highlighted through the realization of optoelectronic and thermoelectric devices with enhanced performance.

## Graphical abstract

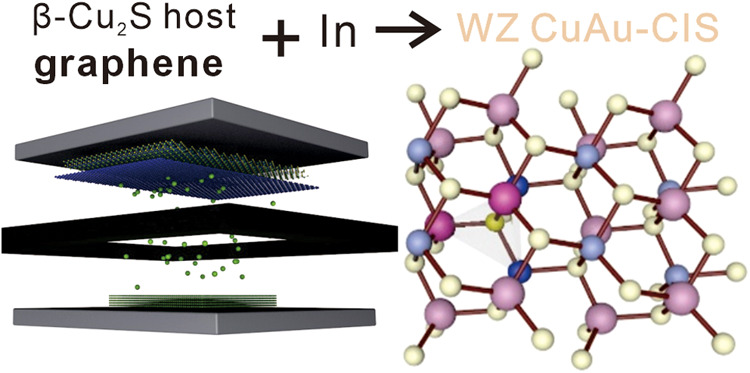

## 1 Introduction

CuInS_2_ is an enabling material for sustainable energy applications due to its combination of favorable electronic, economical, and ecological properties. First, the material boasts a high absorption coefficient,^1^ optimal band gap, and a long carrier lifetime^2^ that make it promising for high-performance solar energy harvesting.^3^ Moreover, its energy levels are well aligned with the requirements for photocatalytic hydrogen production.^4^ Finally, its utilization of nontoxic and low-cost constituents^2^ permits the scaling of the envisioned energy generation devices to industrial relevant dimensions.

Current synthesis methods, however, face significant challenges in achieving sufficiently large and controllable production, thereby limiting the material’s applicability in the envisioned green energy devices. Traditional synthesis approaches, including solution processing, colloidal synthesis, and solid-state reactions, often yield nanocrystals with exceptional properties but on a small scale.^5^ When these nanostructures are integrated into macroscopic devices, their performance degrades due to surface scattering and structural inconsistencies.^2^

Thin film techniques such as sputtering and chemical vapor deposition offer the ability to produce large-scale devices, but they encounter unique challenges specific to CuInS_2_: Due to the complex phase diagram, CuInS_2_ films suffer from the coexistence of multiple crystalline phases.^6^ The resulting mixture of domains produces disorder and non-radiative recombination pathways that compromise the material’s overall performance.^7^ Consequently, a new synthesis route is required that combines the phase stability of low-temperature, wet-chemical synthesis with the scalability of chemical vapor deposition.

In this work, we address these challenges by introducing a novel synthesis approach that yields CuInS_2_ with a singular crystalline phase at the macroscopic scale. We devised an atomic templating strategy that converts a 2D material into CuInS_2_ while retaining the atomic structure imposed by the host. The atomic conversion of a recently discovered transition-metal monochalcogenide^8^ yields metastable wurtzite CuInS_2_ of CuAu type. Structural and spectroscopic analyses confirm the exclusive formation of the CuAu-type wurtzite phase, presenting the first opportunity to characterize its electronic structure. The atomic templating mechanism was elucidated, and a strain-mediated mechanism was proposed and corroborated through ab initio simulations. The layer-by-layer cation-exchange process opens up the unique ability to produce complex ternary 2D material vertical stacks at large scale and without interfacial contamination. We demonstrate the application of this multijunction synthesis process in optoelectronics and thermoelectric devices with high-performance metrics.

## 2 Results and Discussion

The crystalline structure of CuInS_2_ plays a significant role in its optoelectronic properties. A large portion of research has focused on the zincblende-type phase due to its thermodynamic stability.^9^

The CuInS_2_ phase diagram also contains a wurtzite structure that is only stable at elevated temperatures.^9^ The wurtzite phase exhibits a smaller band gap at 1.47 eV and a pronounced pressure tunability of its electronic structure,^10^ which could make it the more attractive crystal phase for energy harvesting applications.

The integration of wurtzite CuInS_2_, however, has been limited due to challenges in its synthesis, as conventional synthesis processes exhibit uncontrollable mixtures of both phases. These issues in producing wurtzite CuInS_2_ are due to the small energy differences between the different phases and subphases, resulting in low selectivity.^6^ Consequently, only nanocrystals with phase mixtures have been realized to date.^11^

To overcome these issues, we pursue an atomic templating process, where a host crystal is modified through the exchange of its constituents. Ion exchange in nanocrystals has demonstrated the impact of the host crystal’s structure on the resulting target crystal.^12^ We extend this concept by introducing 2D materials as hosts due to their known structural robustness^8^ and the demonstrated uniform crystallinity over wafer scale.^13^ The ideal host crystal for wurtzite CuInS_2_ is found to be the 2D transition-metal monochalcogenide β-Cu_2_S. Crystallographic analysis demonstrates that the anion lattice of β-Cu_2_S shows a one-to-one correspondence with the anion lattice of wurtzite CuInS_2_ (Figure 1a).

**Figure 1:**
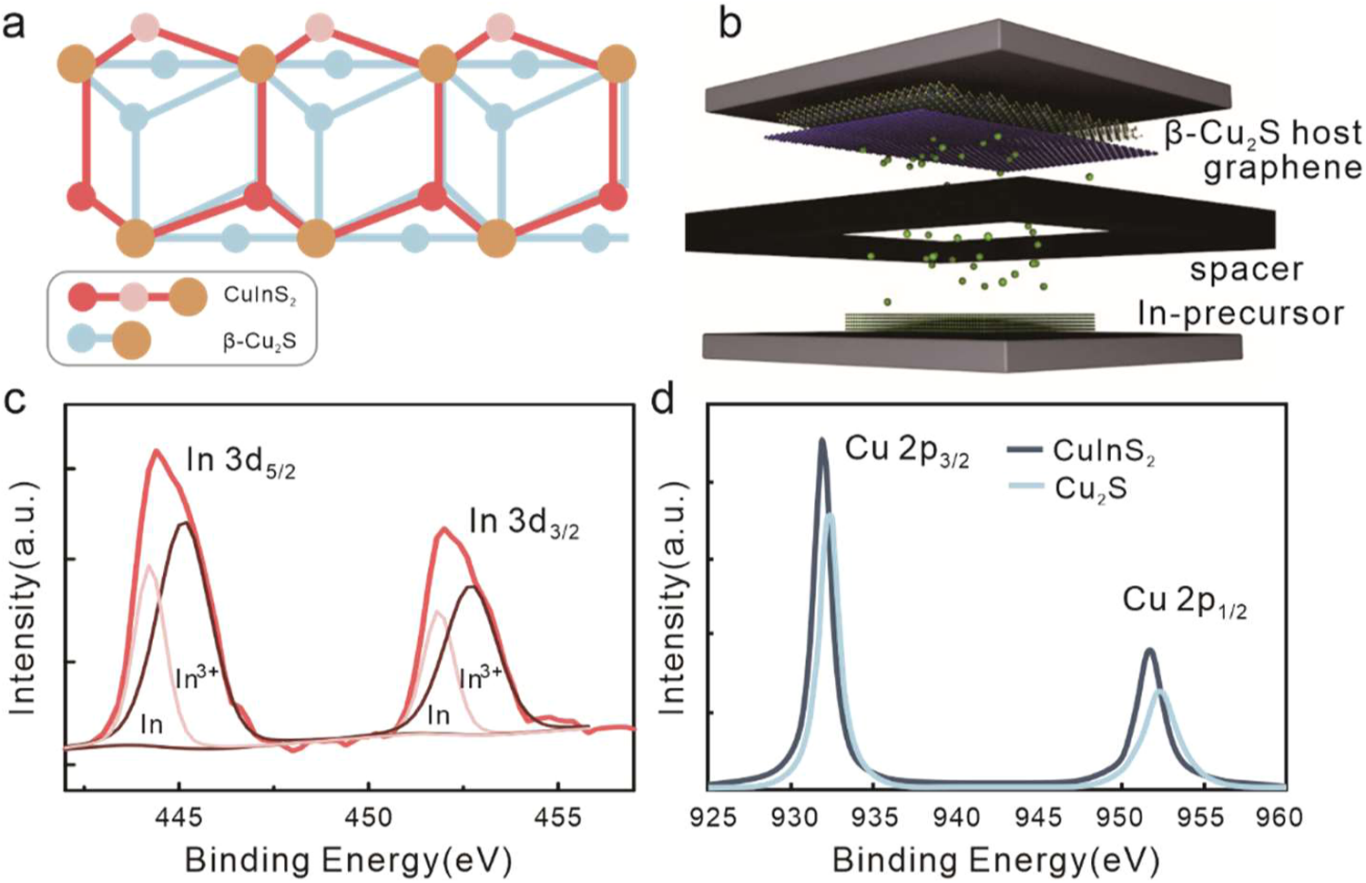
CuInS_2_ synthesis by atomic templating: (a) schematic of atomic templating of CuInS_2_ from β-Cu_2_S showing that the sulfur sublattices of both crystals are identical; (b) depiction of the synthesis process; (c) X-ray photoelectron spectrum of In 3d peak upon deposition, showing the contribution of surface-bound In and bonded In^3+^; and (d) Cu 2p peak showing a peak shift upon indium introduction.

While β-Cu_2_S is a metastable thermodynamic phase, it can be synthesized at large scale using a recently developed confined-reaction approach.^8^ Briefly, graphene serves as a planarization layer that produces a van-der-Waals gap on the surface of a copper substrate. Gaseous sulfur intercalates into the van-der-Waals gap and stabilizes the uncommon hexagonal Cu_2_S phase. This graphene/Cu_2_S structure is transferred from the remaining Cu substrate to SiO_2_/Si substrate following previous reports.^8^

We subsequently transform β-Cu_2_S into CuInS_2_ by exposing it to In vapor. For this purpose, indium was deposited on a Si/SiO_2_ sample and then placed below the graphene/β-Cu_2_S sample with a graphite spacer (Figure 1b). The structure was heated under ambient pressure to 225 °C.

XPS characterization was employed to confirm the incorporation of In into the Cu_2_S lattice. First, the emergence of a sizable indium peak can be seen (Figure 1c). We furthermore observe that the Cu 2p peak shifts to lower binding energies compared to that of pristine Cu_2_S (Figure 1d). This behavior agrees with predictions for the change in binding environments if In is introduced in the Cu_2_S lattice.^10^ The retention of the narrow peak width provides a first indication of the uniformity of the binding environment and suggests that the substantial conversion of Cu_2_S into homogeneous CuInS_2_ occurs.

The conversion of Cu_2_S into CuInS_2_ is expected to yield wurtzite-type crystals due to their structural similarity to the Cu_2_S host crystal. However, wurtzite CuInS_2_ has two polytypes with similar formation energies:^6^ the wurtzite-based CuAu phase (WZ-CuAu symmetry group Pmc21) represents a uniform distribution of Cu and In atoms. The wurtzite-based chalcopyrite (WZ-CH symmetry group: Pna21) shows disordered distributions of cations with either 3 copper or 3 indium atoms surrounding a sulfur site^7^ (Figure 2a).

**Figure 2:**
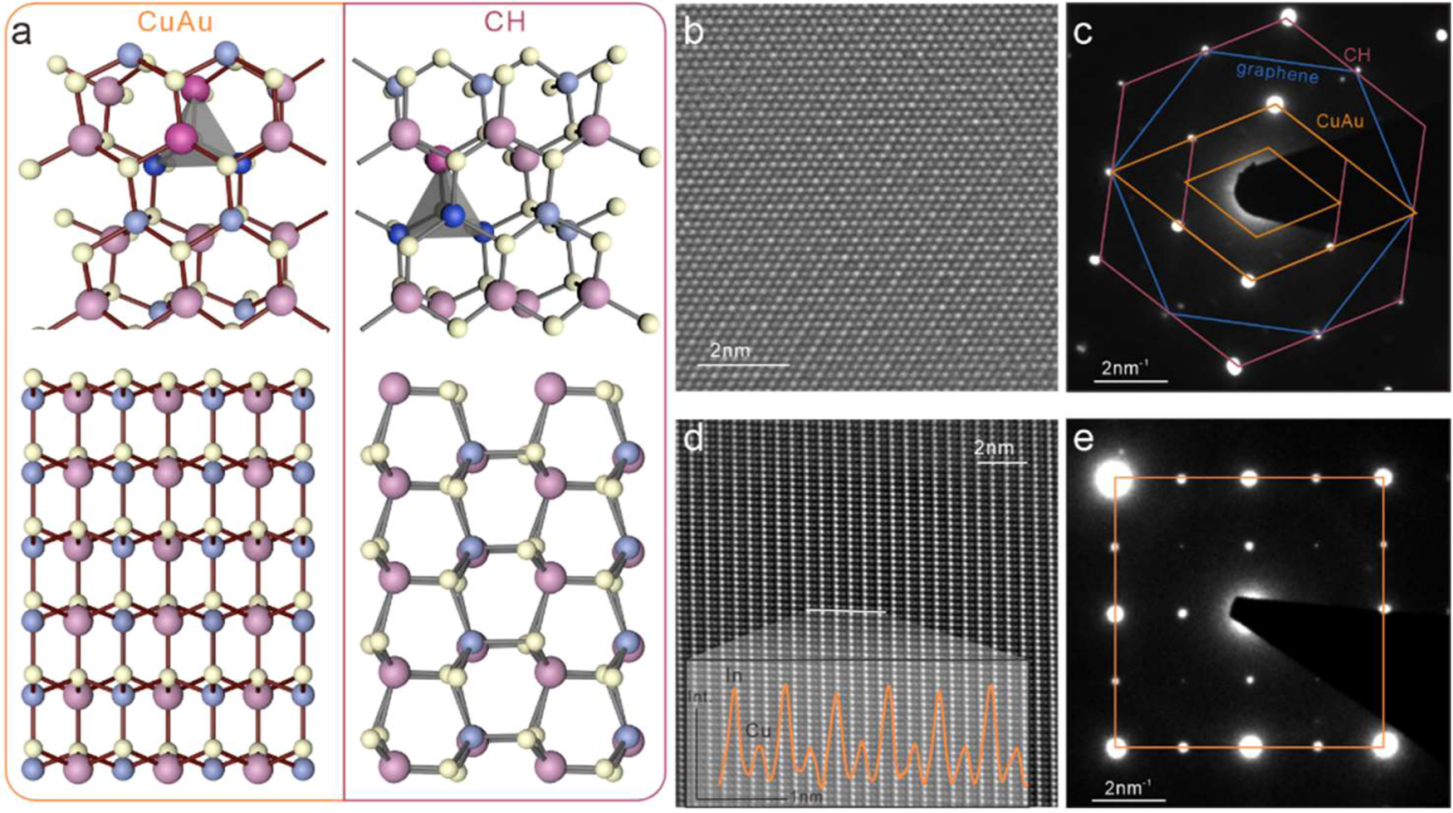
Characterization of the CuInS_2_ structure: (a) depiction of wurtzite chalcopyrite (WZ-CH) and wurtzite CuAu-type (WZ-CuAu) polytypes with indication of a tetragonal unit containing 2Cu (blue)/2In (pink) atoms for WZ-CuAu and 3Cu/1 In for WZ-CH (top) and sideview of both polytypes showing ordered arrangement of Cu (blue) and In­(pink) columns for CuAu; (b) atomic-resolution HAADF STEM image of the basal plane; (c) corresponding selected area electron diffractogram (SAED) with the indication of lattices for graphene, WZ-CH, and WZ-CuAu; (d) HAADF STEM image of the cross-sectional plane showing a columnar arrangement of Cu and In, (inset) z-contrast cross section according to indicated line; and (e) corresponding SAED with the indication of the WZ-CuAu lattice.

To investigate the crystalline structure of the converted material, we conduct atomic-scale electron microscopy. For this purpose, the graphene/CuInS_2_ structure was transferred onto a transmission electron microscopy (TEM) grid. The basal plane exhibits a hexagonal structure (Figure 2b) that could originate from both wurtzite phases and the graphene layer. Selected area diffraction can help distinguish the graphene planarization layer from the crystal (Figure 2c). The remaining reflection spots can be assigned to both the hexagonal or orthorhombic crystal structure (Figure 2c). This ambiguity is due to the weakening of odd lattice reflexes in orthorhombic CuInS_2_ that is enhanced by its epitaxial ordering with graphene.^14^

In order to resolve this issue, we conducted cross-sectional TEM analysis. For this purpose, we converted thick Cu_2_S into CuInS_2_ and searched for folds after transfer. The annular dark-field transmission electron microscopy (ADF-TEM) image shows clear columns of bright and dark atoms when looking at the side of the CuInS_2_ crystal (Figure 2d). This contrast is due to the different scattering efficiencies of heavy In and light Cu, allowing us to distinguish their locations (inset, Figure 2d). This columnar arrangement of atoms agrees with the ac plane of CuAu-type wurtzite and provides clear evidence that wurtzite-type CuAu can be produced through atomic templating, despite its thermodynamic unfavorability. Electron diffraction of the ac plane further confirms the cubic arrangement that rules out the occurrence of the CH-type WZ structure.

To understand the process that leads to the formation of metastable CuInS_2_, we employed time-resolved growth studies, where the extent of CuInS_2_ regions in the Cu_2_S host crystal was analyzed after different growth durations. After short growth times, small grains of CuInS_2_ form within the Cu_2_S 2D material. These seeds are located at the boundaries of the host crystal (inset, Figure 3a). Within 5 min the grains grow and start coalescing into a continuous film.

**Figure 3:**
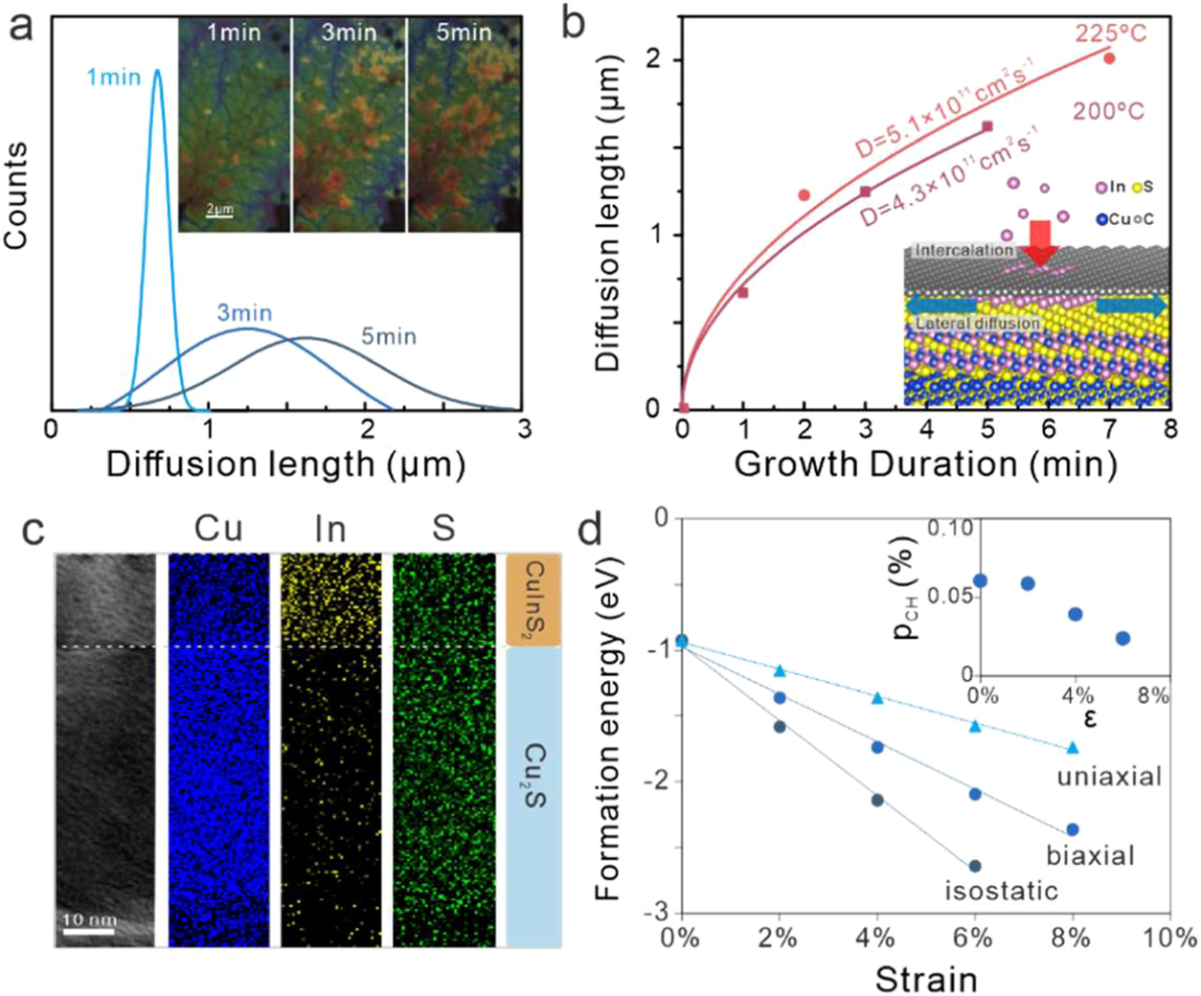
Cation-exchange process: (a) distribution of distances between the converted material and boundary, (inset) optical micrographs of the converted material extent, as visually identifiable by bright regions, after different growth durations; (b) fit of the time-dependent average diffusion length to a diffusion model, (inset) schematic of the proposed cation-exchange mechanism; (c) cross-sectional micrograph of the converted material and the corresponding elemental mapping showing the uniform distribution of Cu and S and abrupt change of In composition; and (d) simulated formation energy per atom vs strain for different strain types in CuAu-type CuInS_2_, (inset) calculated occupation probality (p_CH_) of CH-WZ as a function of strain.

We quantify this observation by tracking the size of multiple grains over time. The distribution of grain sizes broadens with growth time (Figure 3a). This behavior suggests that the conversion process proceeds through diffusion, which is consistent with previously observed cation-exchange processes.^16^ The time evolution of the mean grain size is fitted to a simple diffusion model that shows good agreement with the experimental data. We extract the diffusion coefficient by fitting $\left\langle l\right\rangle =\sqrt{2\mathrm{Dt}}$ and obtain a diffusion coefficient of *D* = 4.3 × 10^–11^ cm^2^ s^–1^. This value agrees well with the surface diffusion coefficient of Au atoms on graphene at the temperature (4.1 × 10^–11^ cm^2^ s^–1^).^17^ From the increase in diffusion coefficient with temperature, we can extract an activation energy of approximately 100 meV. This low value rules out bulk cation transport in Cu_2_S as the rate-limiting step as it exhibits activation energies of ∼500 meV.^18^ Instead, the value is comparable to expected diffusion energies of metals on graphene, which range from 10 meV for Ag to 166 meV for Al,^19^ suggesting that surface diffusion is the controlling process.

Our observation that the templating process is surface-dominated is confirmed by cross-sectional TEM measurements, which show that thick Cu_2_S is retained even after 120 min of annealing and only a 40 nm surface layer is converted (Figure 3c). Moreover, different from lateral diffusion, the in-depth growth yields a sharp interface, as evidenced by compositional mapping (Figure 3c).

Based on these observations, we propose a strain-mediated cation-exchange mechanism (inset Figure 3b). Indium initially permeates the graphene layer through defects and edges to enter the van-der-Waals gap between graphene and Cu_2_S. To achieve the observed epitaxy with the graphene layer, CuInS_2_ has to sustain significant tensile strain. This behavior is corroborated by difference in lattice constants between our diffraction measurements and reported values as well as an upshift of Raman peaks compared to theoretical work as detailed below.^20^ This large interfacial strain limits the motion of indium within the Cu_2_S lattice, and vertical diffusion is suppressed. Consequently, diffusion proceeds mainly parallel to the graphene and produces a planarized growth mode that yields sharp interfaces.^21^

This hypothesis is supported by ab initio simulations (Figure 3d). We utilized LCAO-based density functional theory (DFT) to calculate the formation energy of CuInS_2_ through the exchange of Cu to In atoms in the original Cu_2_S structure. The formation energy is calculated according to

$$E_{\mathrm{form}}=E_{\mathrm{CuIn}S_{2}}^{\mathrm{tot}}-E_{Cu_{2}S}^{\mathrm{tot}}-\left(E_{\mathrm{In}}-E_{\mathrm{Cu}}\right)$$

where the energies represent the total energies of atomic arrangements in the same unit cell and *E*_In_/*E*_Cu_ represents the energies of bare In and Cu within the same unit cell, respectively. We observed that the deformation of the unit cell significantly decreases the formation energy, supporting our strain-mediated growth mechanism. Moreover, our simulations can also explain the observed selectivity toward CuAu-type wurtzite CuInS_2_. Straining increases the formation energy difference between the two crystal structures and thus decreases the probability of producing CH-type CuInS_2_ (inset, Figure 3d). This explanation agrees with predictions of increased energy lowering by straining CuAu-type CuInS_2_ compared to CH due to their smaller Gruneisen parameter.^22^

The observed strain-mediated cationic-exchange mechanism is ideally suited to produce 2D CuInS_2_ that inherits the high crystalline quality and uniformity of the template.^16^ We confirmed this feature by investigating the uniformity of the crystalline phase by spatially resolved Raman spectroscopy.

Due to the homogeneous unit cell, CuAu exhibits a more complex Raman response than chalcopyrite-type CuInS_2_. Indeed, we observe characteristic spectral features at 296 and 307 cm^–1^ that are consistent with f simulations for the LO and TO modes of the A1 symmetric vibration of the WZ-CuAu phase.^6^ Moreover, additional peaks at 240 and 340 cm^–1^ have been previously observed for CuAu-type CuInS_2_ (Figure 4a).^15^ Based on this analysis, the peak intensities around 305 and 295 cm^–1^ can serve as a descriptor of the distribution between CuAu and CH phases: the CH phase exhibits a single LO mode at 295 cm^–1^ but no TO mode, whereas the CuAu phase exhibits both peaks. Consequently, a low peak intensity ratio would correspond to the CH phase, whereas a higher value would represent the LO/TO distribution of WZ-CuAu. We observe a narrow distribution of the 305/295 cm^–1^ intensity ratio with a center of approximately 0.8 (Figure 4b). This value is in quantitative agreement with previous simulations of phase-pure CuAu-type,^6^ indicating the uniform distribution of the material (Figure 4c).

**Figure 4:**
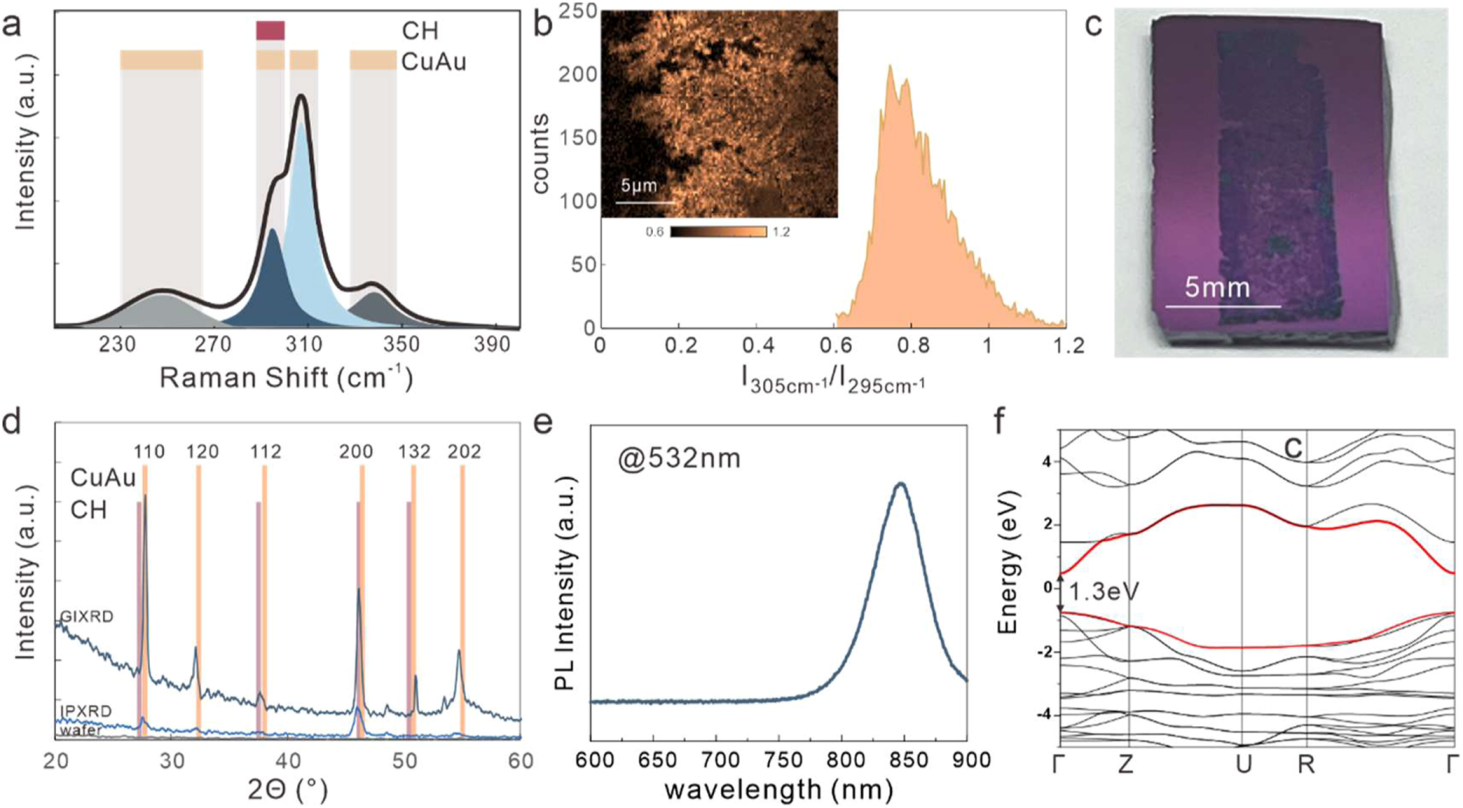
Characterization of uniform CuAu-type CuInS_2_: (a) Raman spectrum with the indication of possible Raman modes for both wurtzite polytypes; (b) histogram of the intensity ratio *I*(ω = 305 cm^–1^)/*I*(ω = 295 cm^–1^) showing the prevalence of CuAu as described in the text, (inset) spatially resolved map of the intensity ratio; (c) photograph of the centimeter-scale sample showing contrast difference between the substrate (300 nm Si/SiO_2_) and grown material; (d) XRD characterization of the material and wafer using grazing angle XRD (GIXRD) and in-plane XRD (IPXRD) with assignment to CuAu modes and indication of possible CH modes; (e) photoluminescence spectrum of the material; and (f) DFT-calculated band structure of CuAu-type CuInS_2_ with the indication of the states closest to the Fermi level and their minimum separation.

This analysis is also supported by an X-ray diffraction analysis. The low thickness of the material makes grazing angle XRD (GIRXD) more effective than conventional XRD as seen in the higher signal-to-noise ratio of the obtained spectra (Figure 4d). The peak assignment shows good agreement with predictions for the CuAu-type CuInS_2_ ^6^ and a reasonable sharpness given the low thickness, which corroborates its high quality. More importantly, a potential co-occurrence of CH-type CuInS_2_ is ruled out as it would produce several overlapping features, but we do not see significant broadening compared to peaks that should not exhibit an overlap. This analysis corroborates our finding that uniform CuAu-type CuInS_2_ can be produced over large scale.

The new capability to produce homogeneous WZ-CuAu-type CuInS_2_ at centimeter dimensions (Figure 4c) permits us to investigate the properties of this metastable material. Previous reports suggested that the CuAu phase is metallic and shows no photoluminescence (PL).^23^ However, our results clearly demonstrate the occurrence of a strong photoluminescence peak at 845 nm (Figure 4e). This result agrees with our DFT calculations that show a direct band gap at the Γ-point (Figure 4f).

Compared to conventional zincblende CuInS_2_, this new phase exhibits a smaller band gap, which enhances its suitability for electronic applications. This property, combined with the material’s scalability and uniformity, positions it as a promising candidate for next-generation optoelectronic devices.

In addition to enabling the realization of unstable thermodynamic phases, the atomic templating process exhibits another advantage. Recent research has demonstrated the promise of 2D material integration within van-der-Waals stacks. Those assemblies are produced by sequential mechanical transfer, which is highly complex and exhibits limited cleanliness and scalability.^24^ Control of the cation-exchange process enables the direct growth of complex 2D material heterostructures. Through sequential growth, three different 2D materials could be stacked without any transfer step. First, graphene growth was conducted on copper foil, which can be achieved at commercially relevant scales and low costs.^25^ In a second step, the graphene/Cu structure was exposed to sulfur to create Cu_2_S by van-der-Waals confined growth, leading to a high-quality graphene/Cu_2_S interface, as previously reported.^8^ The third step represents cationic exchange to CuInS_2_ at a carefully controlled growth duration. This process utilizes the same reactor and can be carried out without breaking vacuum. This direct-growth approach of ternary 2D heterojunctions increases their simplicity, scalability, and commercial appeal. Moreover, the interfaces between the constituents occur through self-organization, which alleviates concerns of contamination.

To illustrate this concept, we demonstrate multispectral Raman maps. Within the same location, the response of CuInS_2_, Cu_2_S, and graphene was characterized (Figure 5a). We find that all materials are continuous. The relative prevalence of Cu_2_S depends on the initial morphology and the processing duration, and we chose a condition that retains large areas of this material in the heterojunction.

**Figure 5:**
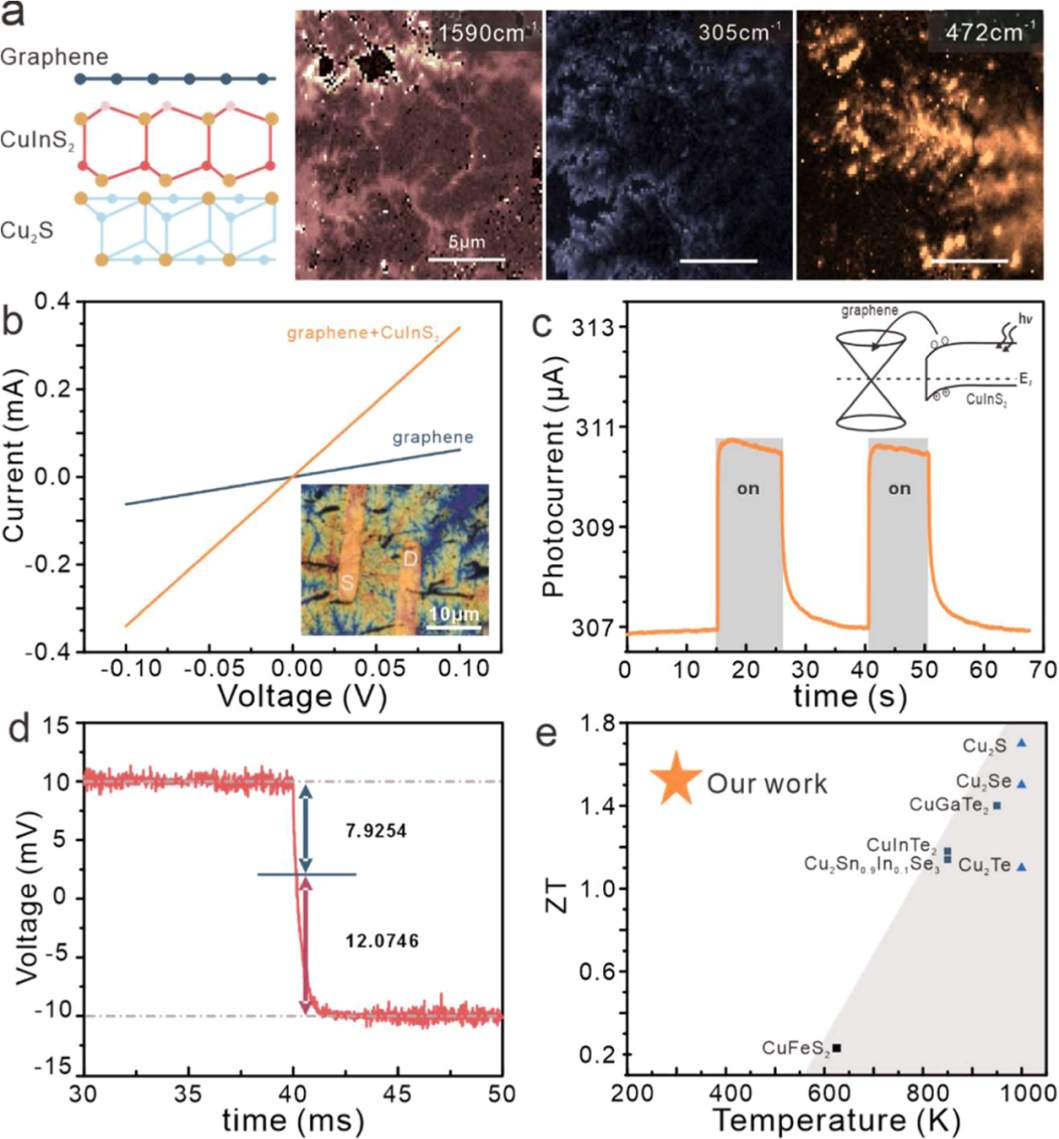
Formation and application of heterostructures: (a) collocated Raman maps of spectral features corresponding to graphene (1590 cm^–1^), CuInS_2_ (305 cm^–1^), and Cu_2_S (472 cm^–1^) that demonstrate the morphology of the graphene planarization layer, the CuInS_2_ middle layer, and the Cu_2_S bottom; (b) current–voltage characteristics of bare graphene device and heterostructure device, (inset) false color optical micrograph of the device; (c) time evolution of the device current under toggled illumination, (inset) schematic of the photoexcited carrier recombination pathway; (d) Harman measurement with the indication of thermal and ohmic voltage components; and (e) comparison of the extracted ZT value with literature values (list in the Supporting Information).

To characterize the resulting triple heterojunction, we conduct carrier transport measurements in a lateral geometry (inset of Figure 5b). We observe that the conductivity of the heterojunction assembly is enhanced over that of the bare graphene control device (Figure 5b). This observation suggests that conduction proceeds efficiently across the heterojunction interfaces and that the underlying layers can assist in the conduction.^26^

We further confirm efficient vertical carrier transport within the heterostructure through optical measurements. For this purpose, a 405 nm laser was toggled on the device and the device current was recorded (Figure 5c). We see that the photocurrent is only 13% of the dark current, which indicates that photoexcited carriers quickly recombine in the graphene (inset, Figure 5c).

The observed low injection barrier in the heterostructure opens up opportunities for thermoelectric devices. Cu_2_S is known to not only exhibit a higher power factor and low thermal conductivity but also exhibits a low electronic conductivity.^27^ Direct combination with graphene could not only alleviate electrical conductivity issues but also increase the thermal leakage. The low predicted thermal conductance of CuInS_2_ ^28^ decouples the two materials and enables their synergistic combination.

Based on this rationalization, we investigate the thermoelectric performance of the graphene/CuInS_2_/Cu_2_S heterostructure in parallel geometry using the Harman method. The thermoelectric voltage is decoupled from the ohmic voltage drop by their time evolution (Figure 5d), and we extract a ZT of 1.52. This value represents the highest reported ZT of copper chalcogenides at this temperature and demonstrates the impact of our approach (Figure 5e).

## 3 Conclusions

In conclusion, we have developed a scalable method for synthesizing the uniform CuAu-type wurtzite CuInS_2_ using a 2D material template. This approach overcomes the challenges of phase disorder and structural inconsistencies in traditional synthesis methods, enabling precise phase control and enhanced material properties. A strain-mediated growth process was proposed that can be leveraged to produce complex 2D material heterostructures with high interfacial quality at large scale. Our findings demonstrate the potential of using a suitable host as an atomic template to stabilize previously unattainable crystal phases, and future work should correlate the structural wealth of 2D materials, such as non-vdW structures^29^ and vdW stacks, as suitable host crystals for complex materials with tailored properties. The promise of this atomic engineering was presented in electronic devices and energy generators.

## 4 Experimental Section

### 4.1 Synthesis and Fabrication

Monolayer graphene was grown on copper foils by LPCVD with CH_4_ (10 sccm) and H_2_ (200 sccm) gases at 1020 °C for 6 h according to previous paper.^30^ β-Cu_2_S was then synthesized with the as-grown graphene/copper at 180 °C in a 1 in. quartz tube, and sulfur powder (Alfa Aesar) is carried by an argon flow following previous reports.^8^

The conversion from Cu_2_S to CuInS_2_ was conducted in a CVD furnace at ambient pressure. Indium (99%, Gredmann) was first coated on the silicon substrate by a thermal evaporator for capping. The chamber was heated up to 225 °C with an argon flow, and the temperature remained constant throughout the conversion process.

### 4.2 Simulation

DFT calculation was carried out by atomistic simulation software QuantumATK^31^ using a numerical LCAO basis set. The hybrid generalized gradient approximation (hybrid GGA) with the HSE06 functional^32^ was used to treat the exchange–correlation interactions in band structure calculation. The relaxation was done with a convergence of force tolerance of 0.05 eV/Å.

### 4.3 Characterization

The as-grown CuInS_2_/graphene structure was transferred from a silicon wafer to TEM grids using NaOH solution. Raman and PL spectroscopy were carried out with a green laser of 532 nm excitation wavelength. TEM was conducted with an FEI Tecnai G2 for SAED, a Spectra 300 FEG-S/TEM for HAADF image, and a JEOL JEM-2100F for cross-sectional EDX mapping.

## Supplementary Material



## Notes

Literature review on the thermoelectric figure of merit for 2D copper-based chalcogenides (PDF)

## References

[1] Chen B., Zheng W., Chun F., Xu X., Zhao Q., Wang F. (2023). Synthesis and hybridization of CuInS_2_ nanocrystals for
emerging applications. Chem. Soc. Rev..

[2] Leach A. D.
P., Macdonald J. E. (2016). Optoelectronic
Properties of CuInS_2_ Nanocrystals
and Their Origin. J. Phys. Chem. Lett..

[3] Braunger D., Hariskos D., Walter T., Schock H. W. (1996). An 11.4% efficient
polycrystalline thin film solar cell based on CuInS_2_ with
a Cd-free buffer layer. Sol. Energy Mater. Sol.
Cells.

[4] Yoon N., Joo O. S., Chae S. Y., Park E. D. (2023). Recent
Advances
in CuInS_2_-Based Photocathodes for Photoelectrochemical
H_2_ Evolution. Nanomaterials.

[5] Long Z., Zhang W., Tian J., Chen G., Liu Y., Liu R. (2021). Recent research
on the luminous mechanism, synthetic strategies,
and applications of CuInS_2_ quantum dots. Inorg. Chem. Front..

[6] Larsen J. K., Sopiha K. V., Persson C., Platzer-Björkman C., Edoff M. (2022). Experimental and Theoretical
Study of Stable and Metastable Phases
in Sputtered CuInS_2_. Adv. Sci..

[7] Qi Y., Liu Q., Tang K., Liang Z., Ren Z., Liu X. (2009). Synthesis
and Characterization of Nanostructured Wurtzite CuInS_2_:
A New Cation Disordered Polymorph of CuInS_2_. J. Phys. Chem. C.

[8] Chin H.-T., Hofmann M., Huang S.-Y., Yao S.-F., Lee J.-J., Chen C.-C., Ting C.-C., Hsieh Y.-P. (2021). Ultra-thin 2D transition
metal monochalcogenide crystals by planarized reactions. npj 2D Mater. Appl..

[9] Park J. S., Dong Z., Kim S., Perepezko J. H. (2000). CuInSe_2_ phase formation during Cu_2_Se/In_2_Se_3_ interdiffusion reaction. J. Appl. Phys..

[10] Xie B.-B., Hu B.-B., Jiang L.-F., Li G., Du Z.-L. (2015). The phase
transformation of CuInS_2_ from chalcopyrite to wurtzite. Nanoscale Res. Lett..

[11] Chang J., Waclawik E. R. (2014). Colloidal semiconductor
nanocrystals: controlled synthesis
and surface chemistry in organic media. RSC
Adv..

[12] Gupta S., Kershaw S. V., Rogach A. L. (2013). 25th Anniversary Article: Ion Exchange
in Colloidal Nanocrystals. Adv. Mater..

[13] Li T., Guo W., Ma L., Li W., Yu Z., Han Z., Gao S., Liu L., Fan D., Wang Z. (2021). Epitaxial
growth of wafer-scale molybdenum disulfide semiconductor single crystals
on sapphire. Nat. Nanotechnol..

[14] Petrović M., zu Heringdorf F. J. M., Hoegen M. H.-v., Thiel P. A., Tringides M. C. (2021). Broad background
in electron diffraction of 2D materials as a signature of their superior
quality. Nanotechnology.

[15] Dzhagan V. M., Litvinchuk A. P., Valakh M. Y., Kruszynska M., Kolny-Olesiak J., Himcinschi C., Zahn D. R. T. (2014). Raman scattering
in orthorhombic CuInS_2_ nanocrystals. Phys. Status Solidi A.

[16] van
der Stam W., Berends A. C., Rabouw F. T., Willhammar T., Ke X., Meeldijk J. D., Bals S., de Mello Donega C. (2015). Luminescent
CuInS_2_ Quantum Dots by Partial Cation Exchange in Cu_2–x_S Nanocrystals. Chem. Mater..

[17] Ruffino F., Cacciato G., Grimaldi M. G. (2014). Surface
diffusion coefficient of
Au atoms on single layer graphene grown on Cu. J. Appl. Phys..

[18] Balapanov M. K., Nadejzdina A. F., Yakshibayev R. A., Lukmanov D. R., Gabitova R. J. (1999). Ionic conductivity
and chemical diffusion in LixCu_2–x_Se superionic
alloys. Ionics.

[19] Tang Y., Zhang H., Shen Z., Zhao M., Li Y., Dai X. (2017). The electronic and
diffusion properties of metal adatoms on graphene
sheets: a first-principles study. RSC Adv..

[20] Kiraci A., Yurtseven H. H. (2017). Calculation
of the frequency shifts and damping constant
for the Raman modes (A_1g_, B_1_) near the tetragonal-cubic
transition in SrTiO_3_. Turk. J. Phys..

[21] Chou Y.-C., Tang W., Chiou C.-J., Chen K., Minor A. M., Tu K. N. (2015). Effect of Elastic Strain Fluctuation on Atomic Layer Growth of Epitaxial
Silicide in Si Nanowires by Point Contact Reactions. Nano Lett..

[22] Wu K., Wang D. (2011). Temperature-dependent
Raman investigation of CuInS_2_ with
mixed phases of chalcopyrite and CuAu. Phys.
Status Solidi A.

[23] Delmonte D., Mezzadri F., Spaggiari G., Rampino S., Pattini F., Bersani D., Gilioli E. (2020). Metastable
(CuAu-type) CuInS_2_ Phase: High-Pressure Synthesis and Structure
Determination. Inorg. Chem..

[24] Lin Y.-C., Lu C.-C., Yeh C.-H., Jin C., Suenaga K., Chiu P.-W. (2012). Graphene Annealing: How Clean Can
It Be?. Nano Lett..

[25] Chin H.-T., Nguyen H.-T., Chen S.-H., Chen Y.-F., Chen W.-H., Chou Z.-Y., Chu Y.-H., Yen Z.-L., Ting C.-C., Hofmann M., Hsieh Y. P. (2021). Reaction-limited
graphene CVD surpasses
silicon production rate. 2D Mater..

[26] Chen T.-W., Hsieh Y.-P., Hofmann M. (2015). Ad-layers
enhance graphene’s
performance. RSC Adv..

[27] Yao Y., Zhang B.-P., Pei J., Liu Y.-C., Li J.-F. (2017). Thermoelectric
performance enhancement of Cu_2_S by Se doping leading to
a simultaneous power factor increase and thermal conductivity reduction. J. Mater. Chem. C.

[28] Giri R. K., Solanki M. B., Chaki S. H., Deshpande M. P. (2023). The DFT
study of thermoelectric properties of CuInS_2_: A first principle
approach. IOP Conf. Ser.:Mater. Sci. Eng..

[29] Chin H.-T., Wang D.-C., Gulo D. P., Yao Y.-C., Yeh H.-C., Muthu J., Chen D.-R., Kao T.-C., Kalbáč M., Lin P.-H. (2024). Tungsten Nitride (W5N6): An Ultraresilient 2D Semimetal. Nano Lett..

[30] Hsieh Y.-P., Shih C.-H., Chiu Y.-J., Hofmann M. (2016). High-Throughput
Graphene
Synthesis in Gapless Stacks. Chem. Mater..

[31] Smidstrup S., Markussen T., Vancraeyveld P., Wellendorff J., Schneider J., Gunst T., Verstichel B., Stradi D., Khomyakov P. A., Vej-Hansen U. G. (2019). QuantumATK:
an integrated platform of electronic and atomic-scale modelling tools. J. Phys.: Condens. Matter.

[32] Heyd J., Scuseria G. E., Ernzerhof M. (2003). Hybrid functionals
based on a screened
Coulomb potential. J. Chem. Phys..

