# Supplementary material for: 2D Atomic Templating for the Large-Scale Synthesis of Metastable CuInS2 and Its Heterojunctions
Source: ACS Appl Mater Interfaces. 2025 Jul 24;17(31):45122–9. doi: 10.1021/acsami.5c07759 (PMC12332815; doi:10.1021/acsami.5c07759)
Supplement: Supplementary file 1 [file am5c07759_si_001.pdf]

Supporting Information for

2D Atomic Templating for the large-scale synthesis of metastable CuInS2 and its heterojunctions

Jui-Teng Chang[1,2], *Yu-Xiang Chen*[2,3,4], *Hao-Ting Chin*[2,3,4], *Ding-Rui Chen*[5], *Jian-Jhang Lee*[2], *Chia-Yi Wu*[6], *Yi-Chia Chou*[6], *Mario Hofmann*[1], *Ya-Ping Hsieh*[2*]

[1] Department of Physics, National Taiwan University, Taipei, 10617, Taiwan

[2]Institute of Atomic and Molecular Sciences, Academia Sinica, Taipei, 10617, Taiwan

[3]International Graduate Program of Molecular Science and Technology, National Taiwan University, Taipei, 10617, Taiwan

[4]Molecular Science and Technology Program, Taiwan International Graduate Program, Academia Sinica, Taipei, 10617, Taiwan

[5]Department of Electronic Engineering, Chung Yuan Christian University, Taoyuan, Taiwan, 32023

[6]Department of Materials Science and Engineering, National Taiwan University, Taipei, 10617, Taiwan

*Corresponding author. Email: yphsieh@gate.sinica.edu.tw

Table S1. Comparison of ZT value for our work with references

Material	Temperature	ZT value	Reference
$CuInTe_2$	850K	1.18	[1]
$CuFeS_2$	625K	0.23	[2]
$CuGaTe_2$	950K	1.4	[3]
$Cu_2Sn_{0.90}In_{0.10}Se_3$	850K	1.14	[4]
$Cu_{2-x}S$	1000K	1.7	[5]
$Cu_{2-x}Se$	1000K	1.5	[6]
Cu_2Te	1000K	1.1	[7]

REFERENCES

(1) Liu, R.; Xi, L.; Liu, H.; Shi, X.; Zhang, W.; Chen, L. Ternary compound $CuInTe_2$: a promising thermoelectric material with diamond-like structure. *Chemical Communications* **2012**, *48* (32), 3818-3820, 10.1039/C2CC30318C. DOI: 10.1039/C2CC30318C.
(2) Xie, H.; Su, X.; Yan, Y.; Liu, W.; Chen, L.; Fu, J.; Yang, J.; Uher, C.; Tang, X. Thermoelectric performance of $CuFeS_{2+2x}$ composites prepared by rapid thermal explosion. *NPG Asia Materials* **2017**, *9* (6), e390-e390. DOI: 10.1038/am.2017.80.
(3) Plirdpring, T.; Kurosaki, K.; Kosuga, A.; Day, T.; Firdosy, S.; Ravi, V.; Snyder, G. J.; Harnwunggmoung, A.; Sugahara, T.; Ohishi, Y.; et al. Chalcopyrite $CuGaTe_2$: A High-Efficiency Bulk Thermoelectric Material. *Advanced Materials* **2012**, *24* (27), 3622-3626. DOI: https://doi.org/10.1002/adma.201200732 .
(4) Shi, X.; Xi, L.; Fan, J.; Zhang, W.; Chen, L. Cu−Se Bond Network and Thermoelectric Compounds with Complex Diamondlike Structure. *Chemistry of Materials* **2010**, *22* (22), 6029-6031. DOI: 10.1021/cm101589c.
(5) He, Y.; Day, T.; Zhang, T.; Liu, H.; Shi, X.; Chen, L.; Snyder, G. J. High Thermoelectric Performance in Non-Toxic Earth-Abundant Copper Sulfide. *Advanced Materials* **2014**, *26* (23), 3974-3978. DOI: https://doi.org/10.1002/adma.201400515.
(6) Liu, H.; Shi, X.; Xu, F.; Zhang, L.; Zhang, W.; Chen, L.; Li, Q.; Uher, C.; Day, T.; Snyder, G. J. Copper ion liquid-like thermoelectrics. *Nature Materials* **2012**, *11* (5), 422-425. DOI: 10.1038/nmat3273.
(7) He, Y.; Zhang, T.; Shi, X.; Wei, S.-H.; Chen, L. High thermoelectric performance in copper telluride. *NPG Asia Materials* **2015**, *7* (8), e210-e210. DOI: 10.1038/am.2015.91.
